# Book Notices and Reviews

**Published:** 1865-05

**Authors:** 


### BOOK NOTICES AND REVIEWS.

*Hand-Book of Skin Diseases.* For Students and Practitioners. By THOMAS HILLIER, M. D., London. With Illustrations. Philadelphia. Blanchard & Lea. 1865.

This is an octavo volume of 353 pages, by one who has had extensive opportunities for observation, as Physician to the Skin department of University College Hospital.

During the last few years our knowledge of cutaneous diseases has been considerably extended, and the author of this moderate-sized book has well performed the labor of furnishing to Students and Practitioners a trustworthy, practical and compendious treatise, which comprises the greater part of what has been long known of cutaneous diseases, and what has been more recently brought to light by English, French, and German dermatologists.

A few original wood engravings have been introduced, to illustrate the microscopical appearances of the hair and cuticle when affected by vegetable growth. The use of the microscope is in the present day almost essential for the diagnosis of some skin diseases, and these drawings, in connection with verbal descriptions, will greatly facilitate the diagnosis of the diseases which they are intended to illustrate.

The Anatomy and Physiology of the Skin are briefly adverted to. In treatment the author has avoided complexity, by not unnecessarily multiplying remedies, and by stating the principles on which treatment should be based.

The work will prove useful to the class for whom it was prepared. For sale by W. B. Keen & Co., 148 Lake st.

---

*Alphabetical Index to Braithwaite's Retrospect.* Embracing parts 1 to 50—1840 to 1865. Comprising twenty-five years of re-publications.

This is a volume of 248 pages, of great value to those who wish to refer to the past numbers of the *Retrospect*; a work which fully reflects the progress of European Medical Science and Literature.

---

*A Vest Pocket Medical Lexicon.* Being a Dictionary of the Words, Terms and Symbols of Medical Science, collated from the best Authorities, with the Addition of New Words not before introduced into a Lexicon, with an Appendix. By D. B. St. John Roosa, M. D., Aural Surgeon to the New York Eye and Ear Infirmary. New York. Wm. Wood & Co., 61 Walker st. 1865.

This little Dictionary is intended to serve as a pocket companion to the student attending Medical Lectures, but by no means as a substitute for the larger works of the same kind. In order to aid in pronunciation the words have been divided into syllables and accentuated. For sale by W. B. Keen & Co., 148 Lake st.

---

*The Pharmaceutists' and Druggists' Practical Receipt Book.* With a Glossary of Medical Terms, and copious Index. By Thomas F. Branston. Lindsay, Blakiston & Co., Philadelphia. 1865.

This is a work of 300 pages, and "is offered to the Chemist, Druggist and Medical Practitioner as a useful manual of reference and information." The Glossary is designed "to assist Students of Medicine in reading old receipts and prescriptions, and to understand the contractions and scientific terms used in the medical art."

